# Evolution of neuropeptide signalling systems

**DOI:** 10.1242/jeb.151092

**Published:** 2018-02-01

**Authors:** Maurice R. Elphick, Olivier Mirabeau, Dan Larhammar

**Affiliations:** 1 School of Biological & Chemical Sciences, Queen Mary University of London, London E1 4NS, UK; 2 Genetics and Biology of Cancers Unit, Institut Curie, INSERM U830, Paris Sciences et Lettres Research University, Paris 75005, France; 3 Department of Neuroscience, Science for Life Laboratory, Uppsala University, Box 593, 75124 Uppsala, Sweden

**Keywords:** Evolution, Invertebrate, Neuropeptide, Phylogeny, Receptor, Vertebrate

## Abstract

Neuropeptides are a diverse class of neuronal signalling molecules that regulate physiological processes and behaviour in animals. However, determining the relationships and evolutionary origins of the heterogeneous assemblage of neuropeptides identified in a range of phyla has presented a huge challenge for comparative physiologists. Here, we review revolutionary insights into the evolution of neuropeptide signalling that have been obtained recently through comparative analysis of genome/transcriptome sequence data and by ‘deorphanisation’ of neuropeptide receptors. The evolutionary origins of at least 30 neuropeptide signalling systems have been traced to the common ancestor of protostomes and deuterostomes. Furthermore, two rounds of genome duplication gave rise to an expanded repertoire of neuropeptide signalling systems in the vertebrate lineage, enabling neofunctionalisation and/or subfunctionalisation, but with lineage-specific gene loss and/or additional gene or genome duplications generating complex patterns in the phylogenetic distribution of paralogous neuropeptide signalling systems. We are entering a new era in neuropeptide research where it has become feasible to compare the physiological roles of orthologous and paralogous neuropeptides in a wide range of phyla. Moreover, the ambitious mission to reconstruct the evolution of neuropeptide function in the animal kingdom now represents a tangible challenge for the future.

## Abstract (precis)

**Summary:** A review of the revolutionary advances in our knowledge of the evolution of neuropeptide signalling systems that have been enabled by comparative genomics and neuropeptide receptor deorphanisation.

## Introduction

The release of peptides as intercellular signalling molecules, which act as neurotransmitters, neuromodulators or neurohormones, is an evolutionarily ancient property of neurons. These ‘neuropeptides’ are derived from larger precursor proteins and are targeted via the regulated secretory pathway to intracellular dense core vesicles, where they are stored until being secreted by exocytosis. Neuropeptides typically exert effects on target cells by binding to and activating specific G-protein-coupled receptors (GPCRs) (see Glossary), leading to changes in the activity of downstream effectors (e.g. enzymes and ion channels). These actions at the cellular level then manifest at the level of organ systems and/or the whole animal as changes in physiological activity and/or behaviour, respectively. Thus, neuropeptide function can be ascribed from the molecular to the behavioural level (Burbach, 2011; Schoofs et al., 2017; Taghert and Nitabach, 2012; van den Pol, 2012).

Glossary **Deorphanisation**

Identification of a ligand that activates an ‘orphan’ receptor (a receptor for which the ligand is unknown).

**Deuterostomes**

A monophyletic branch of bilaterian animals that are phylogenetically distinct from protostomes (see below). Extant deuterostomian phyla include the Chordata, Hemichordata and Echinodermata.

**G-protein-coupled receptor (GPCR)**

GPCRs comprise seven transmembrane domains and when activated by ligands (e.g. neuropeptides) they trigger G-protein-mediated activation or inhibition of downstream effector proteins (e.g. adenylyl cyclase).

**Homologue**

Proteins or genes are homologues if they share sequence similarity that reflects a common evolutionary origin. Homologues can be sub-divided into two types – orthologues and paralogues (see below for definitions).

**Neurophysins**

Cysteine-rich ‘chaperone’ proteins derived from the same precursor proteins as vasopressin/oxytocin-type neuropeptides. Recently, neurophysins were also found in the precursors of ‘NG peptides’, which are paralogues of vasopressin/oxytocin-type neuropeptides in deuterostomian invertebrates.

**Orthologues**

Homologous genes or proteins occurring in different species that evolved from a common ancestral gene/protein through speciation.

**Paralogon**

A set of chromosomal regions comprising syntenic genes that evolved through partial or whole genome duplication.

**Paralogues**

Homologous genes or proteins that evolved by gene duplication in the genome followed by sequence divergence.

**Protostomes**

A monophyletic branch of bilaterian animals that are phylogenetically distinct from deuterostomes (see above). The protostomes comprise two clades – the Ecdysozoa (e.g. arthropods, nematodes) and the Lophotrochozoa (e.g. annelids, molluscs).

**Synteny**

Evolutionary conservation of gene order in chromosomal regions within a species following partial or whole genome duplication or between species following speciation.

**Transcriptome**

The complete set of mRNA transcripts expressed in a cell-type, tissue, organ or organism.

Among the first neuropeptides to be chemically identified in mammals were the hypothalamic neuropeptides vasopressin and oxytocin, which act systemically as hormones (e.g. regulating diuresis and lactation) and act within the brain to influence social behaviour (Donaldson and Young, 2008; Young et al., 2011). Evidence of the evolutionary antiquity of neuropeptide signalling emerged with the molecular identification of neuropeptides in invertebrates – for example, adipokinetic hormone (AKH) and proctolin in insects, and the molluscan cardioexcitatory neuropeptide FMRFamide (Brown, 1975; Price and Greenberg, 1977; Starratt and Brown, 1975; Stone et al., 1976). Furthermore, studies employing antibodies against vertebrate neuropeptides revealed the presence of immunoreactivity in invertebrates (Duve and Thorpe, 1979; Fritsch et al., 1979; Thorndyke and Probert, 1979) and vice versa (Boer et al., 1980). However, because of concerns regarding antibody specificity there was initially uncertainty as to whether the same types of neuropeptides occur in vertebrates and invertebrates (Greenberg and Price, 1983). However, by the late 1980s, definitive proof of the widespread phylogenetic distribution and evolutionary antiquity of some neuropeptides was obtained with the sequencing of ‘vertebrate-type’ neuropeptides isolated from invertebrates (De Loof and Schoofs, 1990). For example, in 1986 a cholecystokinin-type neuropeptide was identified in an insect (Nachman et al., 1986) and in 1987 vasopressin/oxytocin (VP/OT)-type neuropeptides were identified in insect and molluscan species (Cruz et al., 1987; Proux et al., 1987). However, it was not until the turn of the 21st century, with the sequencing of the genomes of the nematode *Caenorhabditis elegans*, the insect *Drosophila melanogaster* and *Homo sapiens* (Adams et al., 2000; *C. elegans* Sequencing Consortium, 1998; Lander et al., 2001), that it became possible to investigate comprehensively the relationships between neuropeptide systems in invertebrates and vertebrates (Hewes and Taghert, 2001; Li et al., 1999b; Vanden Broeck, 2001). Subsequently, neuropeptide ligands for what had previously been referred to as so-called ‘orphan’ GPCRs were identified in *Drosophila* and *C. elegans*, providing fascinating new insights into neuropeptide relationships and neuropeptide evolution (Claeys et al., 2005; Clynen et al., 2010; Hauser et al., 2006; Holden-Dye and Walker, 2013; Husson et al., 2007; Meeusen et al., 2003). For example, it was discovered that the insect neuropeptide AKH is the ligand for a *Drosophila* gonadotropin-releasing hormone (GnRH)-type receptor (Park et al., 2002; Staubli et al., 2002). Subsequently, a GnRH/AKH-like peptide was identified as the ligand for a GnRH-type receptor in *C. elegans* (Lindemans et al., 2009). Thus, a relationship between AKH and GnRH was revealed, and the evolutionary antiquity of GnRH/AKH-type signalling was uncovered (Lindemans et al., 2011).

**Fig. 1.**
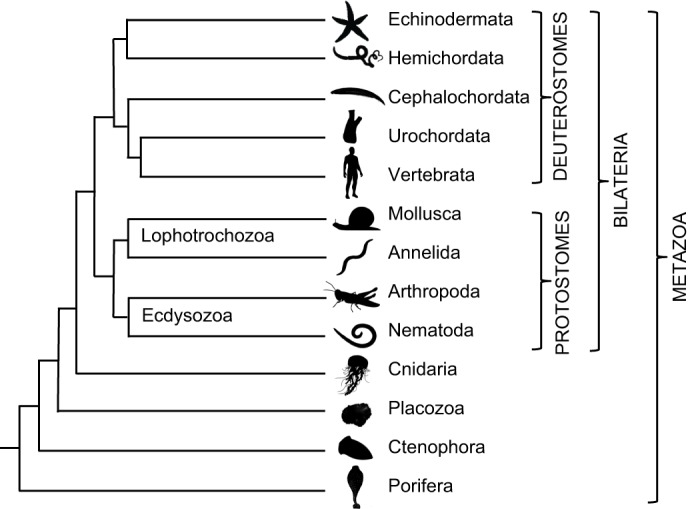
**Animal phylogeny.** Phylogenetic tree showing relationships of selected animal phyla. The Metazoa comprise non-bilaterian phyla and bilaterian phyla. The non-bilaterians include phyla that lack nervous systems (Porifera and Placozoa) and phyla that have nervous systems (Ctenophora and Cnidaria). The bilaterians comprise two super-phyla: the deuterostomes, which include vertebrates, and the protostomes, which include lophotrochozoans (e.g. the mollusc *Aplysia californica*) and ecdysozoans (e.g. the arthropod *Drosophila melanogaster* and the nematode *Caenorhabditis elegans*). Note that the branch lengths in the tree are arbitrary.

Since the *C. elegans*, *Drosophila* and human genomes were sequenced, the genomes of many other invertebrate and vertebrate species have been sequenced, enabling investigation of the evolutionary history of neuropeptide signalling systems. It is our aim here to review the findings of these studies. However, it is first necessary to provide a brief overview of animal phylogeny as a framework for evolutionary interpretations (Fig. 1). Bilaterian animals include two major clades – the deuterostomes and protostomes (see Glossary). Currently, there are only three recognised deuterostomian phyla – the Chordata (which includes vertebrates, urochordates and cephalochordates), the Echinodermata and the Hemichordata. The protostomes comprise many more phyla (>20) and these are grouped in two clades: the Lophotrochozoa (e.g. Mollusca and Annelida) and the Ecdysozoa (e.g. Arthropoda and Nematoda) (Holland, 2011). Investigation of the evolutionary origins of neuropeptide signalling systems has primarily focused on comparison of transcriptome (see Glossary) or genome sequence data from protostomes and deuterostomes. This has enabled identification of a core complement of neuropeptide signalling pathways that can be traced to the bilaterian common ancestor of protostomes and deuterostomes (Jékely, 2013; Mirabeau and Joly, 2013). One bilaterian phylum whose phylogenetic position remains controversial is the Xenacoelomorpha (Cannon et al., 2016; Philippe et al., 2011) and therefore we have not included this phylum in Fig. 1. Nevertheless, investigation of neuropeptide systems in xenacoelomorphs represents a fascinating avenue for future research.

In this Review, we will focus primarily on insights that have been obtained from comparison of neuropeptide systems in protostomes and deuterostomes before moving on to consider how genome duplications have impacted on neuropeptide diversity and function in the vertebrates. However, first we need to consider the pre-bilaterian origins of neuropeptide signalling systems by reviewing findings from non-bilaterian metazoan phyla (also included in Fig. 1).

## Neuropeptide-type signalling systems in non-bilaterian metazoans

There are four known non-bilaterian metazoan phyla: two phyla that have nervous systems, the Ctenophora (comb jellies) and Cnidaria (e.g. sea anemones and jelly fish), and two phyla that lack nervous systems, the Placozoa (e.g. *Trichoplax*) and the Porifera (sponges) (Holland, 2011). It should be noted, however, that evolutionary loss of neurons in the Placozoa and Porifera has not been ruled out (Ryan and Chiodin, 2015). Furthermore, there is controversy regarding the phylogenetic relationships of the Cnidaria, Ctenophora, Placozoa and Porifera (Jékely et al., 2015; Moroz et al., 2014; Pisani et al., 2015). However, the most recent phylogenomic analysis of metazoan phylogeny (Simion et al., 2017) places the Porifera as a sister group to other all other metazoans, with the Ctenophora, Placozoa and Cnidaria occupying the phylogenetic positions illustrated in Fig. 1.

Analysis of genome sequence data has revealed that insulin-type, glycoprotein-type and bursicon-type hormones, which exert their effects by activating leucine-rich repeat-containing GPCRs (LGRs), also occur in non-bilaterian phyla (Roch and Sherwood, 2014), but homologues (see Glossary) of the majority of bilaterian neuropeptides and their cognate receptors have not been found in non-bilaterians (Jékely, 2013). However, a variety of bioactive neuropeptides have been identified in the cnidarians *Renilla köllikeri* (class Anthozoa) and *Hydra magnipapillata* (class Hydrozoa) (Anctil and Grimmelikhuijzen, 1989; Fujisawa and Hayakawa, 2012; Grimmelikhuijzen and Graff, 1986; McFarlane et al., 1991; Morishita et al., 2003; Takahashi et al., 1997, 2008; Yum et al., 1998). Furthermore, sequencing of cnidarian genomes (Chapman et al., 2010; Putnam et al., 2007) has enabled identification of a variety of putative neuropeptide precursors. For example, in the sea anemone *Nematostella vectensis*, precursors of putative neuropeptides with the following C-terminal motifs have been identified: RIamides, RPamides, RWamides, LWamides, ITamide, MTamide, VRamide, RRamide, PGamides, RGamides, PVamides and LVamide (Anctil, 2009). However, none of these neuropeptides appear to be orthologues (see Glossary) of bilaterian neuropeptides.

Sequencing of the genome of the ctenophore *Pleurobrachia bachei* revealed 72 genes encoding putative neuropeptide precursors. However, with the exception of glycoprotein-type, bursicon-type and insulin-type hormones, none of these proteins appear to be orthologues of neuropeptide precursors that have been identified in other metazoans (Moroz et al., 2014). Despite the absence of a nervous system, analysis of the genome sequence of the placozoan *Trichoplax adhaerens* revealed several genes encoding proteins with characteristics of neuropeptide precursors (Nikitin, 2015). Analysis of the genome sequence of a poriferan, the sponge *Amphimedon queenslandica*, has revealed the GPCR repertoire in this species, but homologues of bilaterian neuropeptides and neuropeptide receptors have not been identified (Krishnan and Schioth, 2015; Srivastava et al., 2010).

Interestingly, it has been discovered that several neuropeptides in cnidarians act as ligands for a family of ion channels that are related to mammalian epithelial Na^+^ channels (ENaCs). Thus, the *Hydra* RFamide-type neuropeptides pQWLGGRFamide and pQWFNGRFamide act as ligands for a *Hydra* ENaC-type channel (Golubovic et al., 2007) that is also permeable to Ca^2+^ ions (Dürrnagel et al., 2012). Subsequently, it has been discovered that there is a family of 13 ENaC-type channels that are receptors for *Hydra* RFamide-type neuropeptides (Assmann et al., 2014). Apart from receptors for glycoprotein-type, bursicon-type and insulin-type hormones (see above), neuropeptide-activated GPCRs have yet to be characterised in non-bilaterian animals. There is, however, evidence that they exist (Jékely, 2013) and therefore identifying the ligands for these receptors represents a fascinating area of enquiry for the future. With this perspective in mind, we now move on to consideration of the variety of GPCR-mediated neuropeptide signalling pathways that occur in the Bilateria.

## The evolution and diversity of neuropeptide signalling systems in the Bilateria

### Insights from the genome sequences of *C. elegans*, *Drosophila* and *Homo sapiens*

Sequencing of the genomes of *C. elegans* (Consortium, 1998), *Drosophila* (Adams et al., 2000) and *Homo sapiens* (Lander et al., 2001) provided the first opportunities for comprehensive identification of genes encoding neuropeptide precursors and receptors in bilaterian species, with many interesting new insights being obtained. For example, analysis of the *C. elegans* genome sequence revealed a remarkably expanded repertoire of genes encoding neuropeptides with a C-terminal RFamide motif (Li et al., 1999a,b) and efforts to identify cognate receptors for these peptides are still on-going over 15 years later (Peymen et al., 2014). Analysis of the *Drosophila* genome sequence enabled identification of 44 genes encoding putative G-protein-coupled neuropeptide receptors, with many shown to have orthologous relationships with pharmacologically characterised vertebrate neuropeptide receptors (Hewes and Taghert, 2001; Meeusen et al., 2003; Vanden Broeck, 2001). Subsequently, neuropeptide ligands for many of the receptors in *Drosophila* and other insects have been identified, in some cases revealing unexpected relationships between insect and mammalian neuropeptides (Caers et al., 2012). The example of the GnRH-type AKH receptor has already been given above; another example was the discovery that allatostatin-A type neuropeptides are ligands for galanin-type receptors in *Drosophila* (Birgul et al., 1999; Lenz et al., 2000).

### Insights from the genomes and transcriptomes of other bilaterians

With the application of genome sequencing to species belonging to other bilaterian phyla, additional interesting insights into neuropeptide evolution have been obtained. Thus, sequencing of the genomes of the mollusc *Lottia gigantea* and the annelids *Helobdella robusta* and *Capitella teleta* (Simakov et al., 2013) enabled the first comprehensive analysis of neuropeptide systems in lophotrochozoan protostomes (Veenstra, 2010, 2011) with, for example, a putative homologue of the insect neuropeptide proctolin being discovered in *Lottia*. More recently, detailed analysis of transcriptome sequence data combined with mass spectrometry enabled identification of 98 neuropeptides derived from 53 precursor proteins in the annelid *Platynereis dumerilii* (Conzelmann et al., 2013). Furthermore, a more specific analysis of the occurrence of allatostatin-A/kisspeptin/galanin-related signalling systems in molluscs has also been reported (Cardoso et al., 2016b).

Turning to deuterostomian invertebrates, sequencing of the genome of an echinoderm, the sea urchin *Strongylocentrotus purpuratus* (Burke et al., 2006; Sea Urchin Genome Sequencing Consortium, 2006), provided several new insights into neuropeptide evolution, including the discovery of the first thyrotropin-releasing hormone (TRH)-type precursor to be identified in an invertebrate and the first precursors of pedal peptide/orcokinin-type neuropeptides to be identified in a deuterostome (Rowe and Elphick, 2012).

### 2013 – an annus mirabilis for illumination of neuropeptide evolution

As mentioned above, studies focused on identifying genes encoding neuropeptide precursors and receptors in specific invertebrate species have yielded interesting insights into neuropeptide evolution. However, what was lacking were efforts to integrate data from species belonging to different phyla in a way that would provide a basis for comprehensive reconstruction of the evolutionary history of neuropeptide signalling in the Bilateria. Importantly, two papers published in the *Proceedings of the National Academy of Sciences* in 2013 filled this gap in our knowledge. First, Jékely (2013), employed use of similarity-based clustering methods to investigate evolutionary relationships between neuropeptides and neuropeptide receptors throughout the Metazoa (Jékely, 2013). Second, Mirabeau and Joly (2013) used hidden Markov model (HMM)-based programs and phylogenetic reconstructions to investigate relationships between neuropeptide precursors and receptors in the Bilateria (Mirabeau and Joly, 2013). Here, we highlight some of the key findings from these two papers and then we summarise more recent advances that have been made since 2013.

**Fig. 2.**
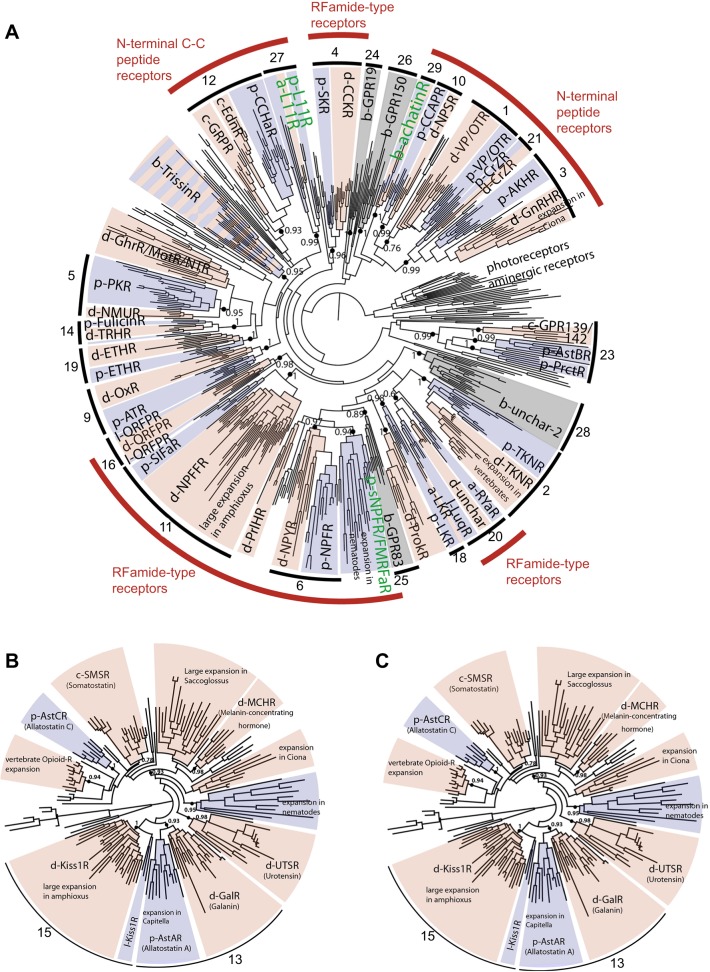
**Phylogenetic analysis of bilaterian rhodopsin-type and secretin-type neuropeptide receptors.** This figure is an updated version of a figure presented previously by Mirabeau and Joly (2013). Maximum-likelihood trees of bilaterian rhodopsin β-type (A), rhodopsin γ-type (B) and secretin-type (C) neuropeptide receptors are shown. The trees contain sub-trees comprising clusters of protostome (blue) and deuterostome (pink) groups of sequences. At the root of blue–pink sub-trees (shown as solid black circles), a prototypic receptor of each subtype was already present in the common ancestor of protostomes and deuterostomes. The bilaterian (b-), protostomian (p-), deuterostomian (d-), chordate (c-), lophotrochozoan (-l) or arthropod (a-) origin is indicated by an initial letter before each peptide GPCR acronym. Trissin receptors are shown with alternating pink and blue stripes because the receptors do not group together in distinct protostomian and deuterostomian clades. Bilaterian clusters where no receptor ligands had been identified by 2013, but which have been identified since 2013 are labelled with green lettering (i.e. elevenin and achatin). Likewise, the post-2013 identification of lophotrochozoan FMRFamide receptors as members of a clade of protostome-specific receptors that include short NPF receptors is also labelled with green lettering. The numbers assigned to each receptor clade correspond to the order in which they are presented in Fig. 3, which provides more-detailed information on the occurrence and characterisation of neuropeptide signalling systems in different taxa. In A, there is additional labelling (outer circle) of groups of receptors that are activated by neuropeptides that share similar characteristics or are derived from precursor proteins that have common characteristics. Photoreceptors and aminergic receptors were used as an outgroup for rhodopsin-β receptors (A), and human adhesion GPCRs were used as an outgroup for the secretin receptors (C).

**Fig. 3.**
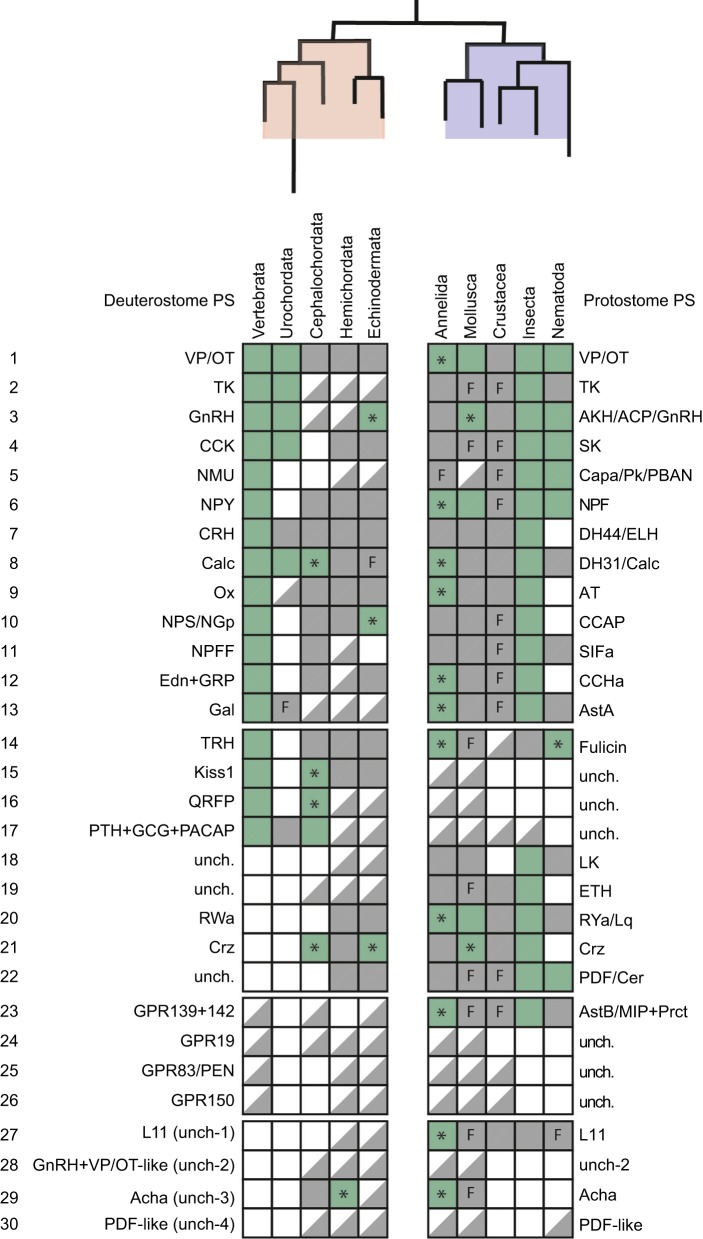
**Occurrence and characterisation of neuropeptide signalling pathways in bilaterians.** This figure is an expanded and updated version of a figure presented previously by Mirabeau and Joly (2013). The occurrence of 30 bilaterian neuropeptide signalling systems in different taxa is shown, with deuterostomian phyla or sub-phyla (pink) shown on the left side, and protostomian phyla/classes shown on the right side (light blue). PS, peptidergic systems. Abbreviated names of neuropeptide signalling systems are used (see Table S1 for full names), which in some cases are the same in all taxa and in other cases can be different; for example, neuropeptide signalling system number 10 comprises neuropeptide-S in vertebrates, NG peptides in deuterostomian invertebrates and CCAP-type neuropeptides in protostomes, which are all ligands for orthologous NPS-, NG peptide- and CCAP-type receptors. A square half-filled with grey indicates that only the receptor of a neuropeptide-receptor signalling pathway has been identified in a taxonomic group. A full grey square indicates that both a peptide ligand and a receptor for a neuropeptide signalling pathway has been identified in a taxonomic group. A full green square indicates that, for at least one member of that taxonomic group, binding between a peptide and its receptor has been demonstrated experimentally. Inclusion of an asterisk in a green full square indicates that binding between a peptide and its receptor has been reported since publication of Mirabeau and Joly (2013). An empty (white) square indicates that a neuropeptide signalling pathway has been lost in a taxonomic group. Inclusion of the letter F in a grey square indicates that experimental insights into the physiological function(s) of the neuropeptide have been obtained. Details of publications that support the conclusions shown here are presented in Table S1.

Mirabeau and Joly (2013) identified 29 bilaterian neuropeptide signalling systems, based on the occurrence of orthologous neuropeptide-type receptors in one or more deuterostomian species and one or more protostomian species. By 2013 neuropeptides that act as ligands for 22 of these receptor types had been identified in at least one species, with the remaining seven receptor types being orphan receptors. Three of these orphan receptors have representation in vertebrates (GPR19, GPR83 and GPR150), whereas the other four are only found in invertebrates (known as bilaterian uncharacterised 1–bilaterian uncharacterised 4, shortened to b-unchar1–b-unchar4). In Figs 2 and 3 we present, in modified form, the major findings of the Mirabeau and Joly (2013) paper together with updates of discoveries that have been made since 2013, which are discussed below.

Of particular interest from the work of Mirabeau and Joly (2013) was the discovery of evolutionary relationships between neuropeptide signalling systems in vertebrates and invertebrates. For example, a relationship between vertebrate orexins and insect allatotropins (Horodyski et al., 2011; Yamanaka et al., 2008) was confirmed based on the identification of a domain in the precursor proteins that is conserved between protostomes and ambulacrarian deuterostomes (hemichordates), but which has been lost in vertebrate orexin precursors. This relationship between orexins and allatotropins was discovered independently by Jékely (2013). Other associations discovered by Mirabeau and Joly (2013) included orthology relationships between: (1) neuropeptide-S-type signalling in vertebrates and crustacean-cardioactive peptide (CCAP)-type signalling in protostomes, (2) neuropeptide FF-type signalling in vertebrates and SIFamide-type signalling in protostomes, and (3) gastrin-releasing peptide/endothelin-type signalling in vertebrates and CCHamide-type signalling in protostomes. Again, two of these associations (1 and 2) were discovered independently by Jékely (2013). Furthermore, another notable finding of Jékely (2013) was the discovery of deuterostomian homologues of the luqin/RYamide-type neuropeptides, which hitherto were only known from protostomes. Likewise, deuterostomian homologues of protostome achatin-type neuropeptides were also discovered. In accordance with the findings of Mirabeau and Joly (2013), Jékely (2013) identified 27 neuropeptide signalling systems that could be traced back to the common ancestor of protostomes and deuterostomes.

### New insights from neuropeptide receptor deorphanisation since 2013

The molecular identity of neuropeptides that act as ligands for bilaterian neuropeptide receptors remain to be identified in many phyla. Furthermore, in some cases nothing is known about receptor ligands in any phyla, as is the case for GPR19 and GPR150 highlighted above, although progress has been made since 2013 with the identification of a neuropeptide ligand for GPR83 (Gomes et al., 2016). Many neuropeptide receptors have been deorphanised (see Glossary) in chordates, insects and nematodes but, until recently, very few neuropeptide receptors had been deorphanised in other taxa. Furthermore, some bilaterian neuropeptide systems first discovered in protostomes and subsequently found in non-chordate deuterostomes were lost in the vertebrate/chordate lineage (e.g. luqin/RYamide). Therefore, gaining a deeper understanding of evolution of these signalling systems will rely on studies that use deuterostomian invertebrates (e.g. echinoderms and hemichordates) as experimental systems.

Since the publication of the papers by Mirabeau and Joly (2013) and Jékely (2013), there have been several studies published that have filled some of the gaps in our knowledge of neuropeptide evolution and neuropeptide relationships in the Bilateria. Most noteworthy is an extensive effort directed at identification of ligands for neuropeptide-type receptors in the annelid *Platynereis dumerilii* (Bauknecht and Jékely, 2015). One of the highlights of this paper was the discovery of a neuropeptide (FSEFLGamide) that is the ligand for a *Platynereis* TRH-type receptor. FSEFLGamide is the first TRH-type neuropeptide to be discovered in a protostome, opening up new opportunities to investigate the evolution and comparative physiology of TRH-type neuropeptide signalling systems in the Bilateria. Accordingly, recent functional characterisation of TRH-type signalling in the nematode *C. elegans* has revealed evidence of an evolutionarily ancient role in the regulation of growth (Van Sinay et al., 2017).

Another highlight of the paper by Bauknecht and Jékely (2015) was the discovery that achatin-type neuropeptides that are present in both protostomes and deuterostomes have an unusual but evolutionarily conserved structural characteristic – the presence of a d-amino acid. Furthermore, phylogenetic analysis of the *Platynereis* and *Saccoglossus* achatin-type receptors (M.R.E., O.M. and D.L., unpublished) reveals that these belong to a clade of receptors that were identified as orphan receptors by Mirabeau and Joly (2013) and designated as ‘b-unchar-3’ (clade 29 in Fig. 2A). These receptors are most closely related to an assemblage of neuropeptide receptors that include receptors for VP/OT-type, NPS/CCAP-type, GnRH-type and corazonin-type peptides, a group of neuropeptides that have the common characteristic of being derived from the N-terminal region of their precursor proteins.

As highlighted above, the molluscan neuropeptide FMRFamide was one of the first invertebrate neuropeptides to be sequenced (Price and Greenberg, 1977). FMRFamide is also present in another lophotrochozoan phylum – the annelids, whereas in ecdysozoan protostomes (e.g. insects) N-terminally extended homologues of FMRFamide or FLRFamide are found (Price and Greenberg, 1989). A *Drosophila* receptor for FMRFamide-like peptides has been identified (Cazzamali and Grimmelikhuijzen, 2002; Meeusen et al., 2002), and Bauknecht and Jékely (2015) reported the identification of a *Platynereis* GPCR that is activated by the tetrapeptide FMRFamide – the first G-protein-coupled FMRFamide receptor to be discovered in a lophotrochozoan. Phylogenetic analysis of the relationship of the *Platynereis* FMRFamide receptor with other bilaterian neuropeptide receptors (M.R.E., O.M. and D.L., unpublished) reveals that it belongs to a family of receptors found only in protostomes and which includes receptors for short neuropeptide F in insects and an expanded family of nematode receptors (Fig. 2A).

A third group of bilaterian neuropeptide receptors that was deorphanised by Bauknecht and Jékely (2015) are the elevenin receptors. The neuropeptide ‘elevenin’ was originally identified in the mollusc *Aplysia californica* on account of its expression in the cholinergic L11 neuron (Taussig et al., 1984). Elevenin contains two cysteine residues that form a disulphide bridge, and related peptides have subsequently been identified in other lophotrochozoans (Conzelmann et al., 2013; Veenstra, 2010, 2011). Furthermore, an elevenin-type signalling system was identified recently in the insect *Nilaparvata lugens* and found to be involved in the regulation of body colour (Uchiyama et al., 2017). Interestingly, phylogenetic analysis reveals that elevenin receptors (branch 27 in Fig. 2A) belong to a group of related GPCRs that are activated by ligands that, like elevenin, typically have one or two disulphide bridges – for example, arthropod CCHamides and vertebrate endothelins (Mirabeau and Joly, 2013) (M.R.E., O.M. and D.L., unpublished).

Use of a lophotrochozoan species (*Platynereis dumerilii*) as a model system for neuropeptide receptor ‘deorphanisation’ (see Glossary), as discussed above, illustrates the importance of research on animals outside the well-studied bilaterian clades (vertebrates and ecdysozoans) to gain insights into the evolution of neuropeptide signalling systems. The ambulacrarians (hemichordates and echinoderms) are of particular interest in this regard because they are an ‘intermediate’ evolutionary lineage with respect to the vertebrates and the protostomes (Fig. 1). An example of this was discussed above with the identification of receptors for achatin-type peptides in the hemichordate *Saccoglossus* (Bauknecht and Jékely, 2015). Next, we will discuss some examples of where deorphanisation of neuropeptide receptors in echinoderms has provided ‘missing links’ for reconstruction of neuropeptide evolution in the Bilateria (Semmens and Elphick, 2017).

As highlighted above, sequencing of the genome of *S. purpuratus* (Sea Urchin Genome Sequencing Consortium, 2006) enabled discovery of the first neuropeptide precursors to be sequenced in an echinoderm (Burke et al., 2006; Elphick and Thorndyke, 2005; Rowe and Elphick, 2010, 2012). Among these sea urchin neuropeptide precursors was a protein with an unexpected characteristic – a neuropeptide precursor comprising two copies of the putative neuropeptide Asn-Gly-Phe-Phe-Phe-NH_2_ (or NGFFFamide) and a C-terminal ‘neurophysin’ domain (Elphick and Rowe, 2009). Hitherto neurophysins (see Glossary) were thought to be uniquely associated with VP/OT-type neuropeptide precursors, where they have a chaperone-like role in targeting VP/OT-type peptides to the regulated secretory pathway (De Bree et al., 2003). The occurrence of a neurophysin domain in the sea urchin NGFFFamide precursor was surprising because NGFFFamide does not have any sequence similarity to VP/OT-type neuropeptides. An explanation for this unusual feature began to emerge with the discovery of a homologous protein in the cephalochordate *Branchiostoma floridae* comprising a C-terminal neurophysin domain and two copies of the putative neuropeptide SFRNGVamide (Elphick, 2010). The NG motif that this peptide shares with NGFFFamide provided a basis for designation of a novel family of neurophysin-associated neuropeptides in deuterostomian invertebrates – the ‘NG peptides’. Furthermore, the discovery that SFRNGVamide is identical to the N-terminal region of a vertebrate neuropeptide known as ‘neuropeptide-S’ (NPS) provided the key clue for determination of the evolutionary history of the neurophysin-associated NG peptides (Elphick, 2010). NPS receptors are closely related to VP/OT-type neuropeptide receptors (Xu et al., 2004). Furthermore, as highlighted above, NPS receptors are orthologues of protostomian receptors for CCAP-type neuropeptides, peptides that exhibit some structural similarities to VP/OT-type neuropeptides (Valsalan and Manoj, 2014). Collectively, this combination of neuropeptide and receptor relationships pointed to a scenario of neuropeptide evolution wherein duplication of an ancestral VP/OT-type neuropeptide signalling system in a common ancestor of protostomes and deuterostomes gave rise to two signalling systems – on the one hand, the highly conserved VP/OT-type signalling system and, on the other hand, the highly divergent NPS/NG peptide/CCAP-type signalling system (Mirabeau and Joly, 2013; Valsalan and Manoj, 2014). Definitive proof of this evolutionary scenario was provided with the discovery that NGFFFamide is the ligand for a NPS/CCAP-type receptor in *S. purpuratus* (Semmens et al., 2015). Thus, an explanation for the presence of a neurophysin domain in the NGFFFamide precursor was obtained. It reflects the retention of an ancient characteristic that is shared with the paralogous neurophysin-containing VP/OT-type precursors, but which has been lost in protostomian CCAP-type precursors and vertebrate NPS precursors (Semmens et al., 2015). However, the functional significance of the retention of the neurophysin domain in the NG peptide precursors of deuterostomian invertebrates remains to be determined.

Analysis of transcriptome sequence data from other echinoderms, including the sea cucumber *Apostichopus japonicus*, the starfish species *Asterias rubens* and *Acanthaster planci*, several brittle star species and the feather star *Antedon mediterrranea*, has enabled identification of many different types of neuropeptide precursors (Elphick, 2012; Elphick et al., 2015; Rowe et al., 2014; Semmens et al., 2016; Smith et al., 2017; Zandawala et al., 2017a). Importantly, several of these precursors have provided new insights into the evolution and diversity of bilaterian neuropeptide signalling systems. For example, the first precursors of kisspeptin-type and melanin-concentrating hormone (MCH)-type neuropeptides to be discovered in a non-chordate species were identified in *A. rubens* (Semmens et al., 2016). Furthermore, the identification of two precursors of GnRH-like peptides in *A. rubens* (ArGnRH1P, ArGnRH2P) provided the basis for new insights into the evolution of GnRH-related signalling, as discussed below.

GnRH was discovered in mammals as a hypothalamic neuropeptide that triggers pituitary release of gonadotropins (Amoss et al., 1971; Schally et al., 1971). As highlighted above, a key breakthrough in our knowledge of the evolution of GnRH signalling was the discovery that AKH is the ligand for a GnRH-type receptor in insects (Park et al., 2002; Staubli et al., 2002). Furthermore, the presence of other GnRH/AKH-like neuropeptides in insects and other arthropods revealed a greater complexity in the diversity of GnRH-related signalling systems. Thus, an AKH-like peptide named corazonin was discovered in cockroaches on account of its excitatory effect on the heart (Veenstra, 1989). Furthermore, when the corazonin receptor was identified it was found to be closely related to GnRH/AKH-type receptors (Cazzamali et al., 2002). A third AKH/corazonin-like neuropeptide was discovered in locusts (Siegert, 1999) and, when an orthologue of this peptide and its cognate receptor were identified in the mosquito *Anopheles gambiae*, the peptide was named AKH/corazonin-related peptide (ACP) (Hansen et al., 2010). However, phylogenetic analysis has revealed that ACP receptors are more closely related to AKH receptors than corazonin receptors (Hauser and Grimmelikhuijzen, 2014).

With the availability of genome sequence data from a variety of invertebrates, it became possible to investigate the evolution of GnRH/AKH/ACP/CRZ-type signalling. Thus, it was discovered that AKH and ACP are paralogous (see Glossary) signalling systems that arose by duplication of a GnRH-type signalling system in a common ancestor of arthropods (Hauser and Grimmelikhuijzen, 2014). Interestingly, orthologues of arthropod corazonin receptors have been found in other protostomes and in invertebrate deuterostomes (Roch et al., 2014a,b), indicating that corazonin signalling is not unique to arthropods but like GnRH-type signalling is a bilaterian signalling system. In accordance with this conclusion, analysis of the transcriptome of the starfish *A. rubens* revealed the presence of transcripts encoding an orthologue of GnRH/AKH/ACP-type receptors (‘ArGnRHR’) and an orthologue of corazonin-type receptors (‘ArCRZR’) (Tian et al., 2016). It was postulated that the ligands for these two receptors are peptides derived from two GnRH-related precursors in *A. rubens* (ArGnRH1P and ArGnRH2P) (Semmens et al., 2016). Heterologous expression of ArGnRHR and ArCRZR revealed that ArGnRH1 and ArGnRH2 are selective ligands for these two receptors, respectively. Hence, ArGnRH1 was designated as ArGnRH, and ArGnRH2 was renamed ArCRZ (Tian et al., 2016). Importantly, this was the first demonstration of the existence of distinct GnRH-type and corazonin-type neuropeptide signalling systems in a deuterostome, providing evidence that the evolutionary origin of these paralogous signalling systems can be traced to the common ancestor of protostomes and deuterostomes (Tian et al., 2016). Interestingly, although GnRH-type signalling appears to have been retained in most phyla, corazonin-type signalling has been lost in several lineages, including vertebrates, urochordates and nematodes. The evolutionary and functional significance of these losses is not yet known, but insights might emerge as we learn more about the physiological roles of GnRH-type and corazonin-type signalling systems in a variety of phyla (Zandawala et al., 2017b).

Before moving on to the vertebrates, we conclude by highlighting research on invertebrates that are the closest relatives of the vertebrates – the cephalochordates and urochordates. Sequencing of the genome of *Ciona intestinalis* (Dehal et al., 2002) enabled the first detailed characterisation of neuropeptides in a urochordate (Hamada et al., 2011; Kawada et al., 2010; Satake et al., 2008; Sherwood et al., 2006). Interestingly, *Ciona* has lost several of the neuropeptide signalling systems that occur in vertebrates and in other invertebrates – for example, it does not have neuropeptide-Y, NPS/CCAP, TRH and kisspeptin (Mirabeau and Joly, 2013). By contrast, the majority of the bilaterian neuropeptide signalling systems are present in the cephalochordate *Branchiostoma floridae* (Mirabeau and Joly, 2013). For example, a QRFP-type signalling system (see below) has been characterised in *B. floridae* (Xu et al., 2015). Furthermore, it was discovered recently that, as with vertebrate calcitonin receptor-like receptors (CLRs), functional expression of a *B. floridae* calcitonin-type receptor requires coexpression of receptor activity-modifying proteins (RAMPs) (Sekiguchi et al., 2016). Kisspeptin-type signalling pathways have also been characterised in *B. floridae*, providing evidence of an evolutionary ancient role for kisspeptins in regulation of reproductive activity (Wang et al., 2017).

## Neuropeptides and their receptors in vertebrates – the impact of genome doublings

**Fig. 4.**
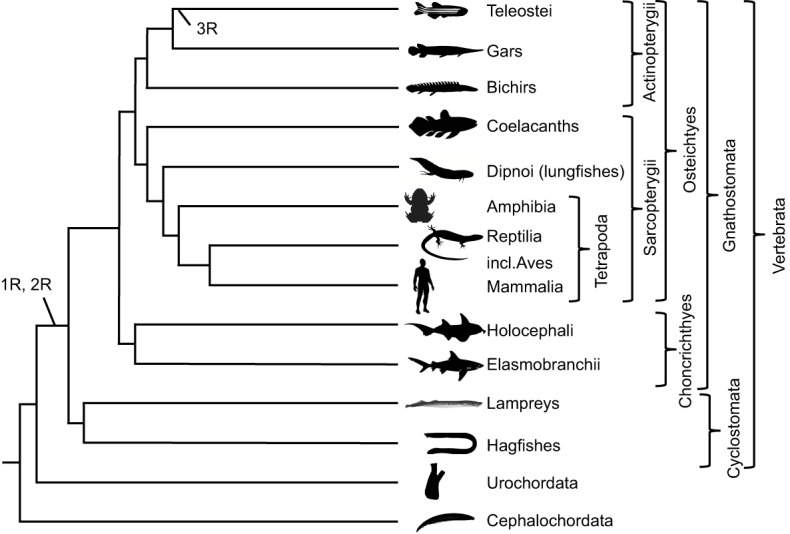
**Chordate phylogeny.** Phylogenetic tree showing relationships of the major chordate lineages. The phylum Chordata comprises three sub-phyla: Cephalochordate, Urochordata and Vertebrata. The vertebrates are sub-divided into the jawless vertebrates (cyclostomes; lampreys and hagfish) and the jawed vertebrates, which are further sub-divided into chondrichthyes (cartilaginous fish) and osteichthyes (bony vertebrates). The bony vertebrates are sub-divided into the Actinopterygii (ray-finned fish) and Sarcopterygii (lobe-finned fish and tetrapods). 1R, 2R and 3R denote first, second and third rounds of genome duplication, respectively.

The origin of the vertebrates coincides with two dramatic events, namely two tetraploidisations called 1R and 2R for the first and second round of genome doubling, respectively (Fig. 4). Both of these took place before the radiation of gnathostomes (i.e. jawed vertebrates) (Nakatani et al., 2007; Putnam et al., 2008). Some evidence suggests that both 1R and 2R took place before the divergence of cyclostomes (lampreys and hagfishes) and gnathostomes at ∼500 Mya, but some investigators argue that these two lineages diverged after 1R. The tetraploidisation events are clearly reflected in quartets of chromosomal regions in gnathostomes in that the four members of such a quartet display similar repertoires of gene families. For instance, the four developmentally important Hox gene clusters are present in four separate chromosomal regions (Pascual-Anaya et al., 2013) that also share members of several other gene families such as voltage-gated Na^+^ channels (Widmark et al., 2011) and insulin-like growth factor-binding proteins (Ocampo Daza et al., 2011). The four related chromosome regions that arose in 2R are said to constitute a ‘paralogon’, a set of related paralogous regions (see Glossary) (Coulier et al., 2000). However, the total gene number is far from quadrupled after 2R owing to extensive gene loss (Holland, 2003). Nevertheless, several hundred, if not thousands, of gene families gained additional members through 1R and 2R. Many neuroendocrine peptide families expanded in 2R, as did a large number of peptide receptor families. Six of these families are described below.

It was initially proposed that gene duplications allow evolution of novel functions – that is, neofunctionalisation (Ohno, 1970) – because it was postulated that, after a gene duplication, one of the copies would maintain the original function, thereby leaving the other copy free to become involved in other processes. This might be more likely if the duplication did not involve all of the regulatory elements of a gene. In contrast, a chromosome duplication (most likely taking place in a tetraploidisation event) means that all the regulatory elements will initially be identical between the two copies. In such a situation, each copy might gradually lose regulatory elements and also expression signals for some cell types, thereby progressively restricting the expression pattern of the two genes. If the two copies lose different elements, they could end up being expressed in separate cell types in a complementary fashion. Such division of the functions of the ‘mother gene’ between the two ‘daughter genes’ has been termed ‘sub-functionalisation’, leading to higher evolutionary plasticity that is referred to as duplication–degeneration–innovation (DDI) (Jimenez-Delgado et al., 2009).

In the ancestor of teleost fishes, a third tetraploidisation called 3R took place ∼350 Mya (Jaillon et al., 2004) (Fig. 4), whereby many families of neuroendocrine peptides and GPCRs expanded further. Consequently, a paralogon in teleosts can consist of up to eight related chromosomal regions, and gene families with eight members, such as the homeobox gene regions and voltage-gated Na^+^ channel genes (Widmark et al., 2011). Other lineages have even had 4R events, such as that of the salmonid fishes ∼100 Mya (Berthelot et al., 2014; Macqueen and Johnston, 2014) and the common carp lineage ∼8 Mya (Xu et al., 2014). All of these lineages can therefore be expected to contain additional copies of genes encoding neuropeptides and their GPCRs.

Here, we do not describe genome duplications beyond 2R, but rather focus on the deduced ancestral repertoire of the vertebrate predecessor before 1R and the deduced setup after 2R, and then describe the present situation in two extant osteichthyans. One is a representative of the actinopterygian (ray-finned) fish lineage, the spotted gar *Lepisosteus oculatus*, which has not undergone 3R. An exception to this is the NPY system, which has not been reported in the spotted gar; therefore, for the NPY system we have included data from another early-radiation osteichthyan, the coelacanth *Latimeria chalumnae*. We also describe the neuropeptides in the tetrapod *Homo sapiens* in detail, as a representative of the lobe-finned fish lineage. The information for cartilaginous fishes still cannot be thoroughly evaluated owing to lack of high-quality genome assemblies, and the same problem occurs for the cyclostome classes of lampreys and hagfishes. The gene repertoires described below have been determined by combining sequence-based phylogenetic analyses with information on conserved synteny (see Glossary) and paralogons.

### Corticotropin-releasing hormone

**Fig. 5.**
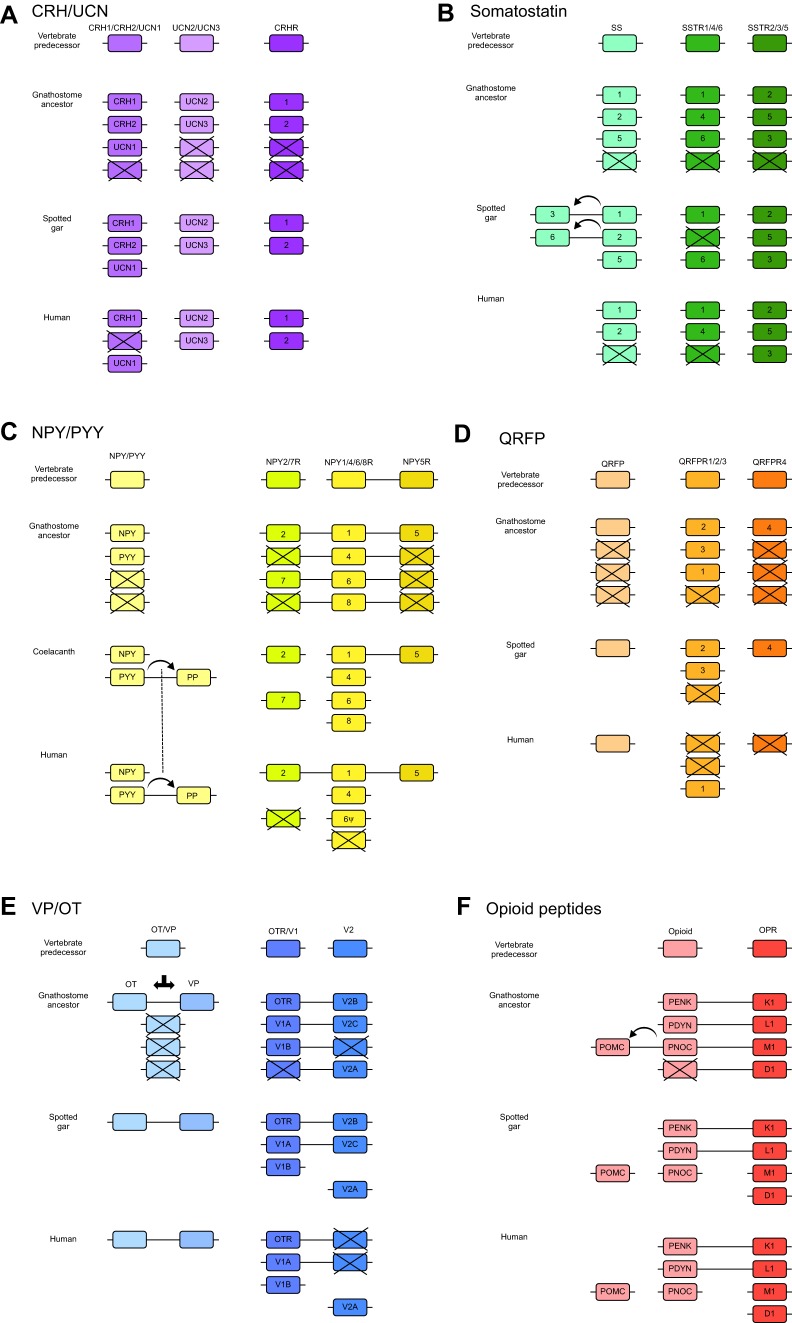
**Impact of genome doublings on six neuropeptide signalling systems in vertebrates.** (A–F) Gene duplications are shown for six peptide–GPCR systems, each including the deduced ancestral single chromosome and the deuterostome predecessor of the vertebrate lineage, followed by the predicted situation in the gnathostome ancestor after the two rounds of genome doubling (2R). Underneath these are two examples of extant species, the spotted gar *Lepisosteus oculatus*, a ray-finned fish representing a lineage that branched off before the teleosts underwent 3R, and *Homo sapiens*. As the NPY system (C) has not yet been reported for the spotted gar, the coelacanth *Latimeria chalumnae* is shown instead because it too has a genome that evolves slowly. Each box corresponds to a single gene. Boxes with crosses denote losses. Genes linked by a line are syntenic. However, there can be several other genes in-between; these have been omitted to highlight the similarities between chromosomes. The ψ symbol for NPY6R in human (C) marks that it has become a pseudogene. A dotted line in C connects the PP duplication in coelacanth and human to mark that this was a single event in their common ancestor. The opioid receptor named L1 in F is the nociceptin (orphanin) receptor, originally named L1 for ‘opioid-receptor-like’. For explanations of other abbreviations and for references, see descriptions in the main text.

Corticotropin-releasing hormone (CRH) is released from the hypothalamus, and stimulates the release of adrenocorticotropic hormone (ACTH) from the anterior pituitary (Smith and Vale, 2006). After the discovery of CRH, additional related peptides were found that brought the number to four in tetrapods, which indicated quadruplication in 2R. However, the members were found to be located in two distinct paralogons, with two peptide genes in each. Very recently, a fifth member of the family, now named CRH2, was found to be part of one of these paralogons and, thus, to have originated in 2R (Cardoso et al., 2016a). Therefore, the two ancestral genes present in the vertebrate predecessor were duplicated in 2R to give rise to one triplet consisting of CRH, urocortin 1 (UCN1) and the newly discovered CRH2, and one pair consisting of UCN2 and UCN3 (Fig. 5A).

The spotted gar has retained all five members (Fig. 5A), as has the coelacanth *Latimeria chalumnae* and the elephant shark *Callorhinchus milii* (Cardoso et al., 2016a), in line with the observation that these species represent lineages that seem to evolve more slowly, both with regard to amino acid sequences and overall genome organisation. The *CRH2* gene was probably lost in the common ancestor of placental mammals. One interesting question is what roles the encoded peptide has in the lineages where it has been retained.

The ancestral CRH/UCN receptor was duplicated in 2R and has been retained in duplicate in all gnathostomes except possibly some cartilaginous fishes (Cardoso et al., 2014). Thus, four to five peptides act through two receptors, giving the opportunity for future research on peptide–receptor preferences in the different vertebrate lineages.

### Somatostatin

Somatostatin was initially discovered in the hypothalamus, where its major role is to inhibit the release of growth hormone from the pituitary (Gahete et al., 2010). Somatostatin was subsequently found to be produced both in endocrine cells in the pancreas and in neurons of the cerebral cortex, as well as elsewhere (Gahete et al., 2010). The somatostatin peptide family consists of three members that arose in 2R (Fig. 5B), of which two remain in tetrapods, called SS1 and SS2 (Tostivint et al., 2013, 2014). The ray-finned fish lineage also retained the third member, SS5, so named as it was characterized after additional members had been identified in teleost fishes. In addition, two local duplicates in the early stages of ray-finned fish evolution have been retained in the spotted gar, bringing its total to five members (Tostivint et al., 2013, 2014).

The somatostatin receptors (SSTRs) are located in two separate paralogons, like the CRH receptors, each of which resulted in triplets after 2R (Ocampo Daza et al., 2012b) – the SSTR1, 4 and 6 subfamily and the SSTR2, 3 and 5 subfamily (Fig. 5B). However, tetrapods and ray-finned fish have lost different members; the tetrapod ancestor lost SSTR6, whereas the ray-finned fish ancestor lost SSTR4, which is the least abundant member of this family in the brain of mammals (Ocampo Daza et al., 2012b; Tostivint et al., 2014). The coelacanth *Latimeria chalumnae* has retained all six of these ancient receptors (Ocampo Daza et al., 2012b). Whether there is a consistent difference in signal transduction between the two SSTR subfamilies remains to be systematically investigated.

### Neuropeptide Y

The two related peptides neuropeptide Y (NPY) and peptide YY (PYY) were discovered in pig brain and intestine, respectively (Tatemoto, 1982; Tatemoto et al., 1982). NPY has been found to influence multiple physiological functions (Pedrazzini et al., 2003), and one of its most prominent roles is in hypothalamic stimulation of food intake (Zhang et al., 2011). Interestingly, PYY released from the intestine has the opposite effect and reduces food intake via the hypothalamus (Batterham et al., 2002). The genes encoding NPY and PYY are located adjacent to homeobox gene clusters A and B, respectively, and were part of the chromosome duplication that generated the Hox cluster quartet, but only two of the post-2R peptide genes remain in gnathostomes (Söderberg et al., 2000; Sundström et al., 2008) (Fig. 5C). The common ancestor of NPY and PYY probably had neuronal expression, because not only NPY but also PYY has neuronal expression in ray-finned fishes (Söderberg et al., 2000; Sundström et al., 2008). Both NPY and PYY have been found in all gnathostomes that have been carefully investigated. All sarcopterygian species that have been investigated also possess a local duplicate of PYY, the pancreatic polypeptide (Larhammar and Bergqvist, 2013).

On the receptor side, the NPY system appears to be the most complex peptide receptor family in the gnathostomes because the ancestral pre-1R chromosome already contained three adjacent receptor genes, the ancestors of the Y1 subfamily, the Y2 subfamily and the Y5 subfamily. After 2R, these subfamilies consisted of four, two and a single member, respectively (Fig. 5C). This ancestral 2R set of seven is still present in the coelacanth *Latimeria chalumnae* (Larhammar and Bergqvist, 2013) as well as in the chondrichthyan elephant shark, *Callorhinchus milii* (Larsson et al., 2009) (the NPY system has not yet been reported in the spotted gar), and is to our knowledge the largest family of rhodopsin-type peptide GPCRs after 2R. In the lineage leading to mammals, two to three of these receptor genes have been lost.

### QRFP

The peptide QRFP begins with the amino acid glutamine (Q) and ends with RFamide, thereby resembling other RFamide peptides and the RYamide peptides NPY and PYY (Elphick and Mirabeau, 2014; Leprince et al., 2017). QRFP was discovered independently in frog and mammalian brain, and a single gene has been found throughout the vertebrates (Leprince et al., 2017). In the hypothalamus, QRFP can potently stimulate food intake (Leprince et al., 2017). A single QRFP receptor has been described in mammals (Fig. 5D), except for rat and mouse, which have a recent duplication (Takayasu et al., 2006). With a wider vertebrate perspective, additional receptor subtypes have been found – first three (Ukena et al., 2014) and then four (Larhammar et al., 2014). Investigation of conserved synteny and paralogons led to the conclusion that the ancestral vertebrate had two adjacent receptor genes, and, after 2R, one was triplicated and the other remained a single gene (Larhammar et al., 2014) (see Fig. 5D).

### Oxytocin/vasopressin

The two related vertebrate peptides oxytocin and vasopressin, which function both as neuropeptides and posterior pituitary hormones (Kiss and Mikkelsen, 2005), did not arise from a common ancestral peptide as a result of 1R or 2R. Instead, their predecessor underwent a local duplication, and the two genes are located close together on the same chromosome in all gnathostomes investigated (Gwee et al., 2008). This duplication took place in the gnathostome ancestor after divergence from the lineage leading to cyclostomes (Gwee et al., 2009).

The receptors for oxytocin and vasopressin have a quite different evolutionary history. A local gene duplication took place before 1R in the deuterostome ancestor of the vertebrates. Subsequently 1R and 2R duplicated this pair so that a total of six genes, two triplets, exist in many vertebrates today (Lagman et al., 2013; Ocampo Daza et al., 2012a), including the spotted gar (Fig. 5E). One triplet consists of genes encoding the oxytocin receptor and the vasopressin V1a and V1b receptors. The other triplet includes the three vasopressin-2 receptor genes V2A, V2B and V2C. Humans today have lost both V2B and V2C. This scenario would seem to explain why the V1A and V1B receptors have a signal transduction mechanism that more closely resembles that of the oxytocin receptor (Ca^2+^ signalling) than that of the V2 receptor (cAMP production). However, the evolution of the signal transduction mechanisms of the different VP/OT receptor subtypes appears to be more complicated (Yamaguchi et al., 2012).

### Opioid peptides

The opioid peptide precursors are more complex than all of the families described above. Endorphin is encoded together with ACTH and melanocyte-stimulating hormone (MSH) in the propeptide called pro-opiomelanocortin (POMC). ACTH and MSH act on a different family of receptors than do opioid peptides. When this propeptide came to encode peptides from two clearly distinct families is still unknown. Enkephalins, dynorphins and nociceptins all come from precursors with multiple opioid-like peptides (Dores et al., 2002; Larhammar et al., 2015; Sundström et al., 2010), and have roles in modulation of pain transmission and in the reward system (Mitsi and Zachariou, 2016). Synteny analyses have shown that the three latter peptide precursors are encoded on three separate chromosomes in the same paralogon (Sundström et al., 2010), and thus probably arose in 2R from a single ancestral peptide gene (Fig. 5F). The *POMC* gene in many species is located in the same chromosomal region as the nociceptin precursor, presumably resulting from a local gene duplication followed by fusion with an *ACTH*/*MSH* gene, although the *POMC* gene has been translocated in both human and spotted gar (Larhammar et al., 2015).

Interestingly, the opioid peptide precursor genes are located in the same paralogon as the four opioid receptor genes (Dreborg et al., 2008). This suggests that the ancestral peptide gene and the ancestral receptor gene were syntenic. However, today any linkage between peptide and receptor genes seems to have been disrupted. Another interesting observation is that all four opioid receptor genes have been retained in all gnathostomes investigated in detail. This is the only gene quartet of those described here, peptide or receptor, that has remained intact throughout the gnathostomes.

### Consequences of the vertebrate genome doublings

Taken together, all of the peptide families, except QRFP, and all of the receptor gene families expanded in the two basal vertebrate genome doublings. Summarising the examples given above, seven pre-vertebrate peptide genes became 14 after 2R (plus local duplications that generated the VP/OT pair and the *POMC* gene before the gnathostome radiation). On the receptor side, 11 genes became 29 after 2R. Thus, it is strikingly obvious that the 2R events expanded the neuroendocrine peptide and GPCR repertoires considerably. For the spotted gar (including coelacanth for the NPY system), 41 of the 43 genes generated in 2R still remain, the only exceptions being SSTR4 and QRFPR1. In humans, only 32 of the 43 original post-2R gnathostome genes have survived. Thus, 11 genes have been lost along the evolutionary lineage to *Homo sapiens*, including the conversion of *NPY6R* to a pseudogene.

Four of the six vertebrate peptide families described above have invertebrate homologues: CRH is related to DH44 in arthropods, somatostatin is related to allatostatin C, NPY is related to NPF, and VP/OT has invertebrate homologues (Mirabeau and Joly, 2013). These homologies are further supported by the observation of receptor similarity between vertebrates and invertebrates. QRFP binds to a receptor in the extended NPY receptor family. By contrast, the sixth peptide family described above, the opioid peptides, has no apparent invertebrate homologue, not even among invertebrate deuterostomes. Nor do the opioid receptors have any closely similar homologues among invertebrates. Thus, the opioid system of peptides and receptors appears to be a vertebrate novelty. Another alternative is that this peptide–receptor system existed in deuterostomes before 1R, but has been lost in the non-gnathostome lineages. In addition, the ACTH/MSH peptides mentioned above as parts of the POMC precursor seem to have arisen at the origin of the vertebrates.

The functional roles of many of the gene duplicates still remain to be characterized in detail, including how functions, distribution and abundance may differ between vertebrate lineages. Knowledge about their evolutionary relationships will help formulate the most interesting questions to address. Furthermore, genome duplication is not unique to the vertebrate lineage because there is evidence that it has also occurred in some invertebrate lineages, including bdelloid rotifers (Flot et al., 2013) and arachnids (Schwager et al., 2017). Accordingly, analysis of genome sequence data from arachnids and other chelicerates has revealed the existence of paralogs for many neuropeptide precursors and receptors (Veenstra, 2016). These taxa will provide interesting material for comparative analysis of the impact of whole-genome duplication on the evolution and physiological roles of neuropeptide signalling systems.

## General conclusions and looking ahead

The availability of genome/transcriptome sequence data from an ever-growing diversity of invertebrate and vertebrate species has facilitated reconstruction of the evolution of neuropeptide signalling systems in the animal kingdom. Thus, the evolutionary origin of at least 30 neuropeptide signalling systems can be traced back to the common ancestor of protostomes and deuterostomes, but with numerous examples of lineage-specific loss. Neuropeptide orthologues in different phyla are often highly divergent, which can make determination of neuropeptide relationships based only on comparison of neuropeptide sequences very difficult. Thus, it has been the identification of receptors that mediate the effects of neuropeptides that has been crucial in determining the orthology of representatives of neuropeptide families in different phyla. Using this approach, unexpected relationships have been revealed, enabling detailed reconstruction of the evolutionary history of neuropeptide signalling pathways (e.g. the neuropeptide-S, NG peptide, CCAP neuropeptide family; Semmens et al., 2015). However, there still remain many gaps in our knowledge of the evolution and diversity of neuropeptide signalling in the Bilateria (Fig. 3). For example, ligands for leucokinin-type receptors have yet to be identified in deuterostomes, whereas probable peptide ligands for GPR19 and GPR150, and receptors for pedal peptide/orcokinin-type peptides have yet to be identified in any phyla. Furthermore, there are many bilaterian phyla in which transcriptomic/genomic-level analysis of neuropeptide signalling are just beginning to be explored (e.g. Onychophora, Priapulida and Tardigrada; Christie et al., 2011) or have yet to be explored (e.g. Xenacoelomorpha, Nemertea, Brachiopoda, Phoronida, Entoprocta, Rotifera and others).

The pre-bilaterian origins of the bilaterian neuropeptide signalling pathways are also unknown. To the best of our knowledge, neuropeptides that act as ligands for GPCRs have yet to be identified in any of the four non-bilaterian phyla – Porifera, Placozoa, Ctenophora and Cnidaria. If GPCRs that mediate the effects of neuropeptides or neuropeptide-like molecules in non-bilaterian phyla can be identified, novel insights into the evolutionary origins of the bilaterian neuropeptide signalling systems could be obtained.

There are still many missing pieces in the ‘jigsaw puzzle’ of neuropeptide evolution. However, the progress that has been made over the last decade or so has been remarkable. Not so long ago the diversity of neuropeptides in the animal kingdom appeared to be represented as an order-less collection of largely unrelated molecules. But now we have the core framework of neuropeptide evolution reconstructed and we can look forward to integrating into this framework functional insights. There is a rich history of research investigating the physiological/behavioural roles of neuropeptides in the animal kingdom, and the article by Jékely et al. (2018) highlights some of the key insights that have been obtained. Furthermore, advances in transcriptomics and peptidomics are enabling profiling of neuropeptide expression in identified populations of neurons in multiple species [see review by DeLaney et al. (2018) in this issue]. With the availability of such high-resolution data, there are emerging opportunities to explore the evolution of neuropeptide function at multiple levels, from identified neurons to networks of synaptically (and non-synaptically) connected populations of neurons to whole-animal physiology and behaviour.

## Supplementary Material

Supplementary information
