# Supplementary material for: Evolution of neuropeptide signalling systems
Source: J Exp Biol. 2018 Feb 1;221(3):jeb151092. doi: 10.1242/jeb.151092 (PMC5818035; doi:10.1242/jeb.151092)
Supplement: Supplementary information [file jexbio-221-151092-s1.pdf]

Table S1

Click here to Download Table S1
